# Supplementary material for: Inhibition of Liver Tumor Cell Metastasis by Partially Acetylated Chitosan Oligosaccharide on A Tumor-Vessel Microsystem
Source: Mar Drugs. 2019 Jul 13;17(7):415. doi: 10.3390/md17070415 (PMC6669685; doi:10.3390/md17070415)
Supplement: Supplementary file 1 [file marinedrugs-17-00415-s001.pdf]

Supporting Information

Inhibition of Liver Tumor Cell Metastasis by Partially Acetylated Chitosan Oligosaccharide on A Tumor-Vessel Microsystem

Bolin Jing [1,2], Gong Cheng [1], Jianjun Li [1,*], Zhuo A. Wang [1,*] and Yuguang Du [1,*]

[1] State Key Laboratory of Biochemical Engineering, Institute of Process Engineering, Chinese Academy of Sciences, Beijing 100190, P.R. China

[2] University of Chinese Academy of Sciences, Beijing 100049, P.R. China

* Correspondence: jjli@ipe.ac.cn (J.L.); wangzhuo@ipe.ac.cn (Z.A.W.); ygdu@ipe.ac.cn (Y.D.); Tel.: +86-10-8254-5070 (J.L.&Z.A.W.&Y.D.)

Supplementary information:

Figure S1. The operating schematic of the human tumor metastasis microsystem.

Figure S2. MTT assay. Statistical analysis the viability of HepG2 cells treated by COS, paCOS (F_A = 0.46), and NACOS at the concentration of 100 μg/mL for 24 h. 5-Fu (100 μg/mL) was used as a positive control. Data are represented as the means ± SD (n = 8), *P < 0.05, **P < 0.01, ****P < 0.0001.

Figure S3. Inhibitory effects of paCOS with F_A 0.46 on liver tumor cells proliferation. Statistical analysis of the proliferation rate of SMMC-7721 cells (A) and MHCC97-L cells treated by paCOS with F_A 0.46 at different concentrations dissolved in culture medium on the tumor-vessel microsystem for 24 h. 5-Fu (100 μg/mL) was used as a positive control. Data are represented as the means ± SD (n = 5), **P < 0.01, ****P < 0.0001.

Figure S4. Inhibitory effects of paCOS with F_A 0.46 on the viability and permeability of endothelial cells (EAhy926). Statistical analysis of the viability (A) and the P_{app} value (B) of EAhy926 cells treated by paCOS at different concentrations dissolved in culture medium on the microfluidic chip for 24 h. 5-Fu (100 μg/mL) was used as a positive control. Data are represented as the means ± SD (n = 5), ** P < 0.01, *** P <0.05, **** P < 0.0001.

Figure S5. Inhibitory effects of paCOS with F_A 0.46 on liver tumor cells invasion. HE staining views (scale bar: 100 μm) (A) and statistical analysis (B) of the invasion rate of HepG2 cells treated by paCOS with F_A 0.46 at different concentrations dissolved in culture medium on Transwell system for 24 h. 5-Fu (100 μg/mL) was used as a positive control. Data are represented as the means ± SD (n = 5), **P < 0.01, ***P <0.05, ****P < 0.0001.

Figure S1

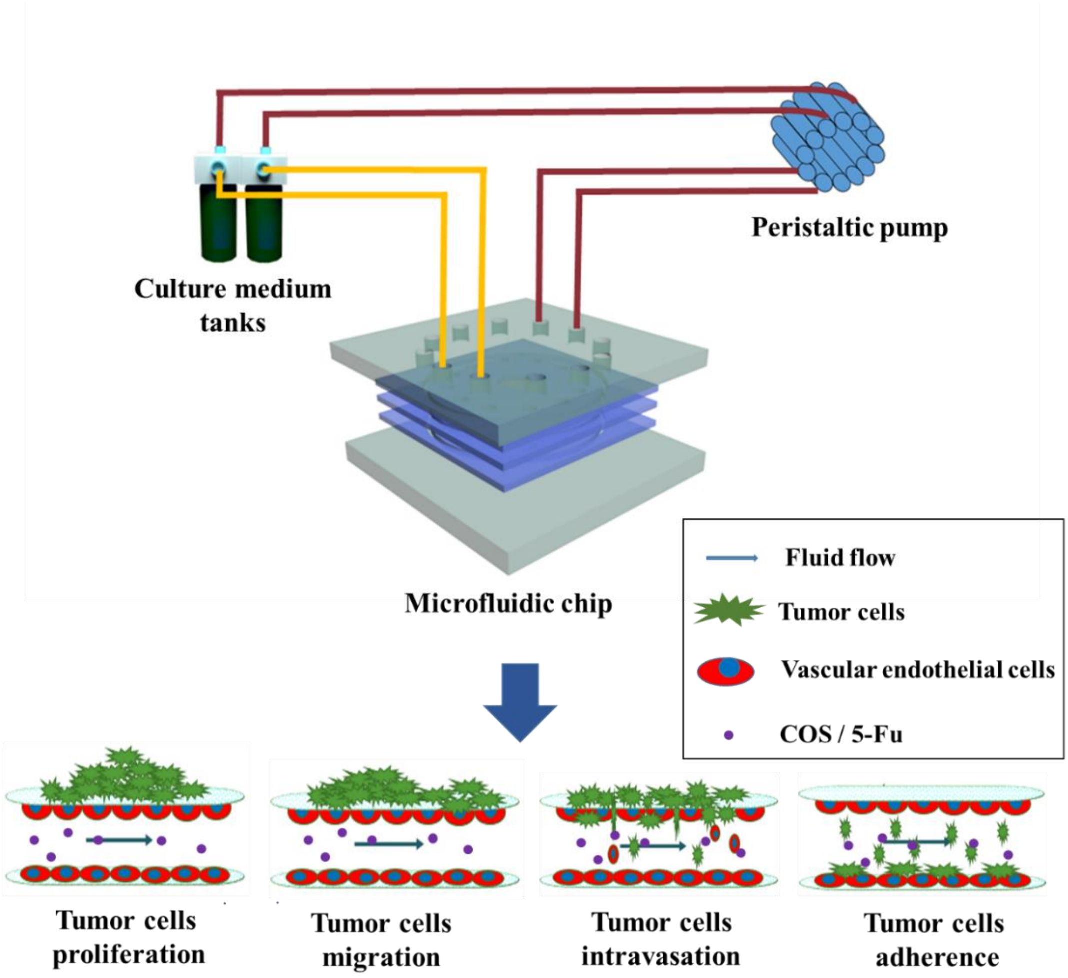

Peristaltic pump
Culture medium tanks
Microfluidic chip
Fluid flow
Tumor cells
Vascular endothelial cells
COS / 5-Fu
Tumor cells proliferation
Tumor cells migration
Tumor cells intravasation
Tumor cells adherence

Figure S2

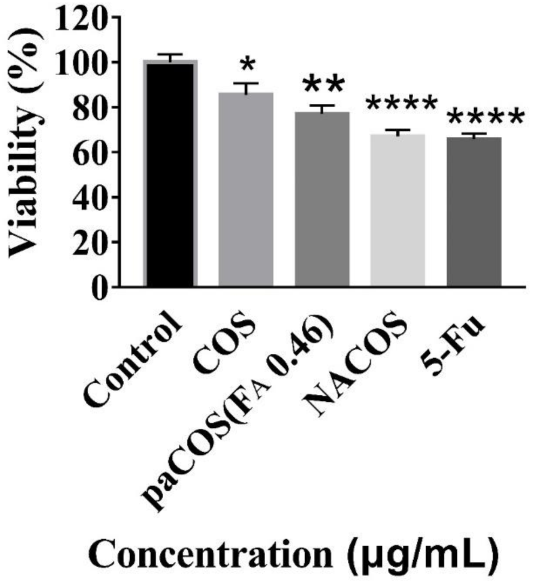

Viability (%)
120
100
80
60
40
20
0
*
**
****
****
Control
COS
paCOS(FA 0.46)
NACOS
5-Fu
Concentration (μg/mL)

Figure S3

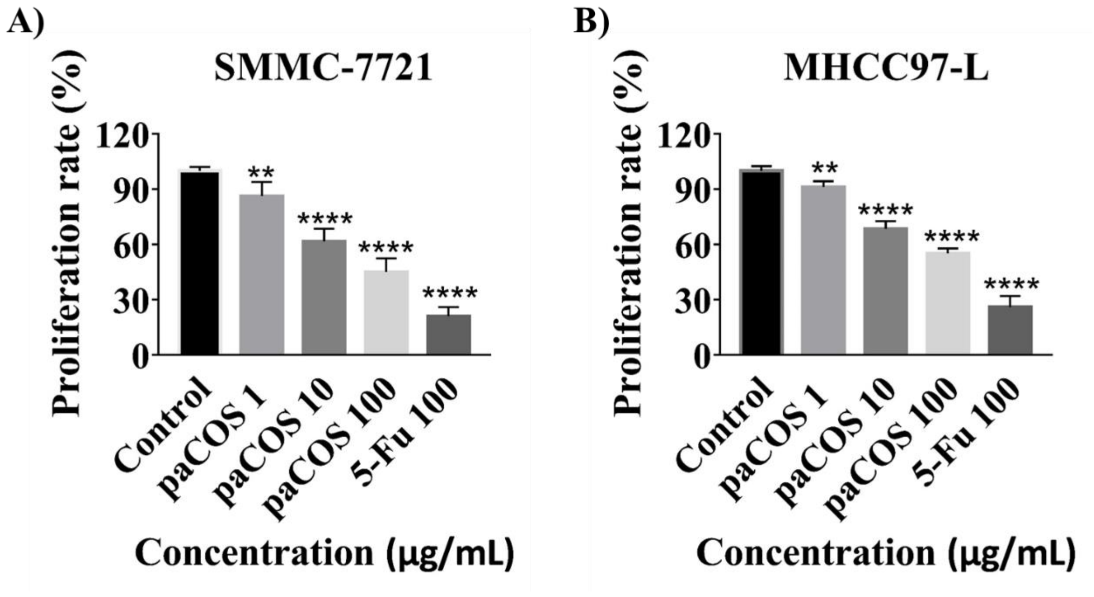

A)
SMMC-7721
Proliferation rate (%)
120
90
60
30
0
**
****
****
****
Control
paCOS 1
paCOS 10
paCOS 100
5-Fu 100
Concentration (μg/mL)
B)
MHCC97-L
Proliferation rate (%)
120
90
60
30
0
**
****
****
****
Control
paCOS 1
paCOS 10
paCOS 100
5-Fu 100
Concentration (μg/mL)

Figure S4

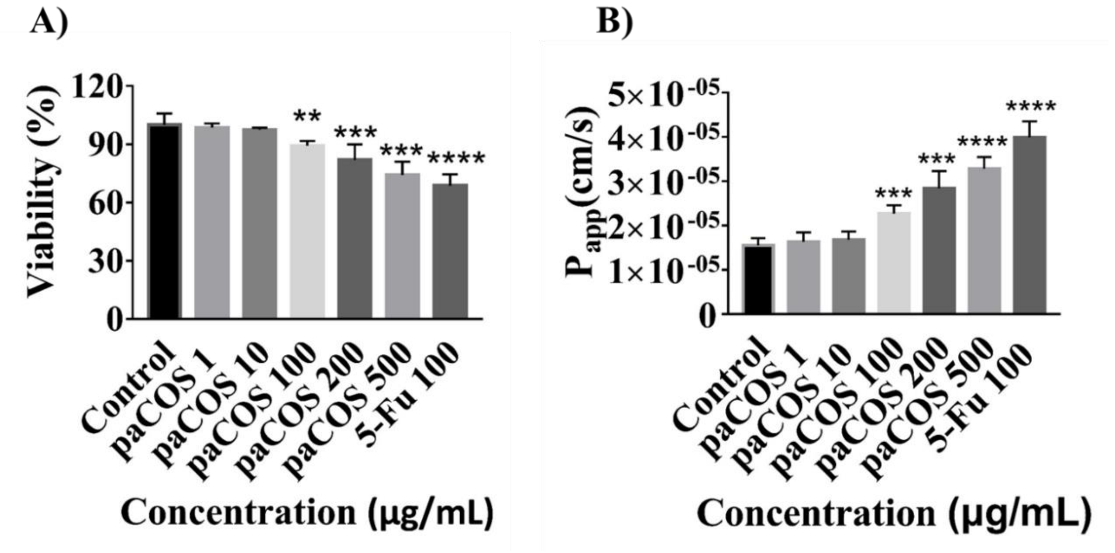
A)
Viability (%)
120
90
60
30
0
**
***
***
****
Control
paCOS 1
paCOS 10
paCOS 100
paCOS 200
paCOS 500
5-Fu 100
Concentration (μg/mL)
B)
P_app(cm/s)
5×10^-05
4×10^-05
3×10^-05
2×10^-05
1×10^-05
0
***
***
****
****
Control
paCOS 1
paCOS 10
paCOS 100
paCOS 200
paCOS 500
5-Fu 100
Concentration (μg/mL)

Figure S5

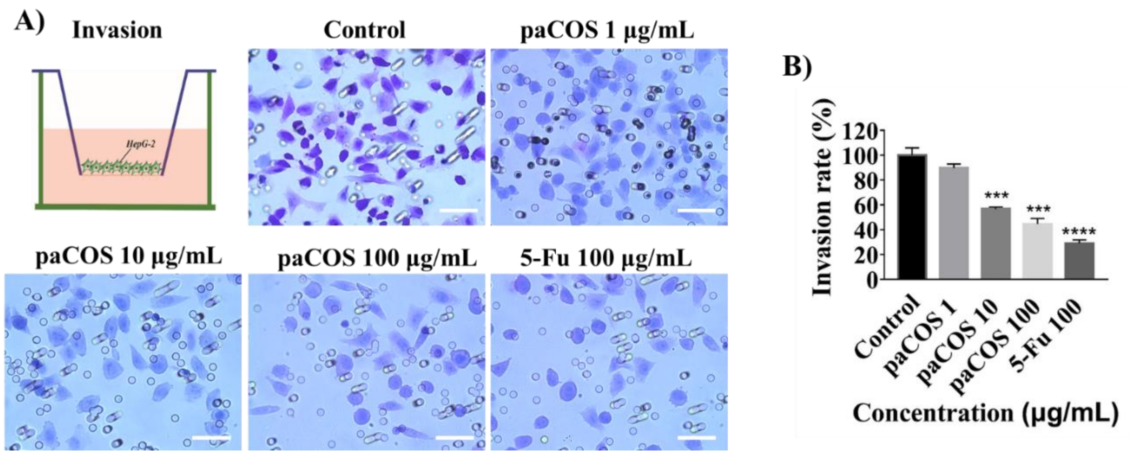
A)
Invasion
HepG-2
Control
paCOS 1 μg/mL
paCOS 10 μg/mL
paCOS 100 μg/mL
5-Fu 100 μg/mL
B)
Invasion rate (%)
120
100
80
60
40
20
0
***
***
****
Control
paCOS 1
paCOS 10
paCOS 100
5-Fu 100
Concentration (μg/mL)
